# Click-through Rate Prediction and Uncertainty Quantification Based on Bayesian Deep Learning

**DOI:** 10.3390/e25030406

**Published:** 2023-02-23

**Authors:** Xiaowei Wang, Hongbin Dong

**Affiliations:** College of Computer Science and Technology, Harbin Engineering University, Harbin 150001, China

**Keywords:** CTR prediction, feature interaction, Bayesian deep learning, uncertainty quantification

## Abstract

Click-through rate (CTR) prediction is a research point for measuring recommendation systems and calculating AD traffic. Existing studies have proved that deep learning performs very well in prediction tasks, but most of the existing studies are based on deterministic models, and there is a big gap in capturing uncertainty. Modeling uncertainty is a major challenge when using machine learning solutions to solve real-world problems in various domains. In order to quantify the uncertainty of the model and achieve accurate and reliable prediction results. This paper designs a CTR prediction framework combining feature selection and feature interaction. In this framework, a CTR prediction model based on Bayesian deep learning is proposed to quantify the uncertainty in the prediction model. On the squeeze network and DNN parallel prediction model framework, the approximate posterior parameter distribution of the model is obtained using the Monte Carlo dropout, and obtains the integrated prediction results. Epistemic and aleatoric uncertainty are defined and adopt information entropy to calculate the sum of the two kinds of uncertainties. Epistemic uncertainty could be measured by mutual information. Experimental results show that the model proposed is superior to other models in terms of prediction performance and has the ability to quantify uncertainty.

## 1. Introduction

With the rise of multimedia applications, CTR prediction tasks have appeared in many scenarios, such as precise advertising placement and product recommendations on e-commerce platforms. Since every user’s click behavior can be regarded as a display of their preferences, CTR prediction is the process of learning user preferences from stored historical user data and then using them to predict future behavior.

The development of CTR prediction models can be divided into two aspects: automatic feature engineering and improving model capabilities. Since the features in CTR prediction are usually in specific fields, how to effectively learn rich information from feature interaction becomes a key challenge of CTR prediction. For the early CTR prediction model, due to the limitation of computing capacity, artificial feature engineering and a simple machine learning model were adopted. The method used in the CTR prediction task was linear models, such as the logistic regression (LR) [1] model, which has the advantage of simplicity and efficiency, but the disadvantage is that the model is very dependent on feature design. Some models that emerged later, such as factorization machine (FM) [2], attentional factorization machine (AFM) [3], etc., can learn the intersection information of the two features. Since the application of feature engineering in CTR prediction largely relies on the experience of experts. Therefore, it is an important research orientation of CTR prediction to design new models to automatically realize feature combinations to explore the hidden information hidden in feature interactions.

Subsequently, significant progress has been made in CTR prediction due to advances in deep neural networks (DNNs) in learning representations. Models, such as the product-based neural network (PNN) [4], deep cross network (DCN) [5], and factorization-machine based neural network (DeepFM) [6] can obtain higher-order feature interactions. A lot of work has been carried out in recent years to combine explicit feature interaction with DNN. However, these methods suppose all features interact and that the interaction of each feature should be modeled to the same extent. While deep interest network (DIN) [7], deep interest evolution network (DIEN) [8], and JointCTR [9] combine historical user behavior to perform a personalized interest model. The powerful function of deep learning is that it can construct complex functions and perform nonlinear transformations on data [10]. All of the above methods only focus on important feature interactions of second or higher-order, while ignoring the importance of each feature to the predicted target [11]. Worthless features yield noise and complicate the feature interaction procedure. Therefore, in feature learning, there are a few methods to learn feature importance, such as feature importance and bilinear feature interaction network (FiBiNET) [12], and dual input-aware factorization machine (DIFM) [13]. However, deep learning usually cannot solve the uncertainty problem in actual application scenarios.

Because of the limitation of available training samples and the existence of incoherent noise, quantification uncertainty should be an integral part of any prediction system. While the development of machine learning tools means large progress, the uncertainty in the models maintains a huge gap worth exploring. In essence, most existing research is based on deterministic models and is short of the capability to acquire uncertainty. As we all know, Bayesian theory is commonly used to describe modeling errors and also provides mathematical tools for the uncertainty of inference models. Deep learning and Bayesian statistical theory are combined as Bayesian deep learning to offer uncertainty estimates for deep structures. In contrast to traditional deep learning models that produce deterministic predictions, Bayesian machine learning provides not only predictions but also uncertainty, which is estimated by the probability density of the outcome.

Gal et al. [14] proposed a simpler method for deep learning uncertainty estimation by training a dropout network and using dropout to obtain predicted Monte Carlo (MC) samples at test time. Optimizing neural networks with dropout can be equated to a type of variational inference in Bayesian machine learning models. A Bayesian machine learning model determines the uncertainty of the input sample model by predicting the variance of the output probability distribution. The output samples are regarded as MC samples that are extracted from the posterior distribution of the model by employing standard dropout to the DNN during the test phase. To achieve MC, a DNN needs to be trained by dropout. Second, to calculate the inference for each input, the DNN method of dropout is applied at T times during the test phase. This theory has been successfully applied in wind energy, power grids, climate prediction, and other fields, proving that the method can capture the uncertainty related to the model output. Inspired by these, we propose a CTR prediction model composed of feature selection and feature interaction. In this framework, uncertainty is divided into epistemic uncertainty and aleatoric uncertainty, which respectively explains the uncertainty caused by model parameters and structure and data noise.

The Innovations of this work can be summarized as:

(1) We propose a CTR prediction model based on Bayesian deep learning, which learns high-order feature interaction information and models of two kinds of uncertainties. The model achieves efficient and reliable prediction.

(2) A framework combining feature selection and feature interaction is proposed. In the feature selection module, CNN and MLP are used to generate meaningful feature interactions. The feature interaction part is modeled by squeeze network and DNN parallel for feature interactions, respectively.

(3) In the above framework, the Monte Carlo dropout is used to perform a posteriori approximate inference on the parameters of the model and obtain the integrated prediction results. The uncertainty of the model is quantified by information entropy.

(4) Experiments on three datasets evidence that the prediction performance of our proposed model performs better than the most advanced models. A reliable uncertainty quantification is carried out for the model.

The remainder of our work is made up of the following: Section 2 introduces related work. Section 3 describes the proposed model. In Section 4, detailed experimental consequents and analyses are introduced. Finally, we summarize the work of this paper in Section 5.

## 2. Related Works

(1) CTR Prediction

In recent years, CTR prediction tasks have developed rapidly and have made very big breakthroughs. Its research mainly uses machine learning methods to predict the user’s clicks on advertisements in subsequent times based on the historical behavior data of users and attribute information.

Initially, some shallow models were used. The combination of the LR model and artificial feature engineering, such as mixture of logistics regression (MLR) [15], gradient boost decision tree + logistic regression (GBDT + LR) [16], etc., are widely exploited. Its advantage lies in its efficiency and ease of deployment. Then, the FM model realizes a pair-to-pair combination of features. Following the idea of the FM model, a series of upgraded models are optimized for different emphases, such as field-aware factorization machine (FFM) [17], higher-order factorization machine (HOFM) [18], field-weighted factorization machine (FwFM) [19], and multi-order interactive features aware factorization machine (MoFM) [20].

Factorization machines deep neural network (FNN) [21] was the earliest proposed model based on deep learning. The PNN model introduced the product layer in the neural network and had two feature cross calculation methods of inner product and outer product. The wide and deep learning (WDL) [22] model adopted a dual-path structure, which joined the generalization of the deep model with the memory of the wide linear model. DeepFM was a combination of neural networks and FM, which could simultaneously model high- and low-order features. The DCN model proposed a CrossNet to explicitly learn finite-order feature crossovers, and implicitly learn crossover features through DNN. Extreme deep factorization machine (xDeepFM) [23] proposed a compressed interaction network (CIN) for vector-wise feature interaction that could obtain explicit and implicit high-order feature interaction simultaneously. Sina proposed the FiBiNET model, adopted the squeeze-and-excitation network (SENET) to learn the importance of features, and learn feature interactions by a bilinear function. Liu et al. [24] introduced a neural attention network to study the significance of second-order interaction and employed two different residual networks to explore feature interactions automatically.

The DIN model is the first method to introduce attention to user behavior modeling, which assigns different weights to users according to their historical behavior and the relevance of current goods through the attention module. On this basis, the DIEN and deep session interest network (DSIN) [25] models are derived. The automatic feature interaction learning (AutoInt) [26] model was drawn on the multi-head self-attention mechanism of the transformer in the natural language processing (NLP) model, and the weights obtained simultaneously give the model a certain degree of interpretability. The DIFM utilized fully-connected layers and multi-head self-attention networks, respectively, to obtain feature interactions from bit-wise and vector-wise levels. The dual-view attention network (DVAN) [27] proposed a selection mechanism to draw feature interactions, respectively, from the item- and user-view. The attentive capsule network (ACN) [28] model employed transformers for feature interaction and adaptively seized multiple interests from user behavior history using capsule networks.

(2) Uncertainty in the Models

Deep learning models with uncertainty measurements have been widely used in many applications. For example, Wang et al. [29] proposed an ensemble probabilistic prediction learning system to quantify the uncertainty of crude oil prices by combining five commonly used machine learning methods with an improved optimizer. Gal et al. proposed dropout as an approximate Bayesian inference of network weights and demonstrated that dropout could be utilized to apply Bernoulli distributions to the weight of convolutional network filters in the test phase, to assess model uncertainty and prediction. In subsequent studies, Liu et al. [30] combined variational Bayesian inference and spatial-temporal neural networks to predict spatial-temporal wind speed. Sun et al. [31] combined Bayesian probability theory and deep long short-term memory (LSTM) for grid load prediction research. Xiao et al. [32] used neural network structures to quantify model uncertainty and data uncertainty for various NLP tasks. Hernández and López [33] applied Bayesian deep learning to quantify the uncertainty of plant disease detection. Ghoshal et al. [34] proposed a Bayesian CNN based on weights for estimating uncertainty in the COVID-19 chest X-ray dataset to increase the performance of diagnostics. Abdar et al. [35] designed a Bayesian neural network model for biomedical image segmentation to quantify uncertainties in classification. Wang et al. [36] modeled the uncertainty through Bayesian deep learning to improve personalized recommendations. Zheng et al. [37] presented semantic uncertainty to explain incomplete data acquisition and inaccurate data labeling. Jin et al. [38] proposed a variational Bayesian deep prediction network with a self-screening layer to solve the problem that a large amount of noise and data conflict or inconsistency reduces the prediction accuracy. They have successfully demonstrated the practical significance of technology in model prediction and uncertainty estimation.

## 3. Methods

### 3.1. Problem Description

(1) CTR prediction. Given training data D=(X, Y). $X={\left\{x_{i}\right\}}_{i=1}^{N}$ are the input data, the corresponding outputs $Y={\left\{y_{i}\right\}}_{i=1}^{N}$, where Y is a set of class labels for binary classification, representing the click behavior for a given ad. The target of the CTR prediction is to construct a model $\hat{y}=CTR\;Model\left(X\right)$ that predicts the probability P(y|x;θ) that the user clicks on the recommended advertisement, where θ is the parameter of the model;

(2) The epistemic uncertainty is a measure of the uncertainty of the model parameters. It derives from the limited representation ability of the observed value, which denotes the difference between the predicted value $\hat{y}$ and the true value y. It is quantified by mutual information;

(3) The aleatoric uncertainty measures the noise intrinsic in the data. This uncertainty cannot be eliminated. It is expressed as the output of a function, which is represented by the difference between the predicted entropy and mutual information;

(4) Objective function. Logloss is regarded as the loss function for a binary classification problem. We train our model by minimizing the following objective function:

(1) $$logloss=-\frac{1}{N}\overset{N}{\underset{i=1}{\sum }}\left(y_{i}log\left({\hat{y}}_{i}\right)+\left(1-y_{i}\right)log\left(1-{\hat{y}}_{i}\right)\right)$$

where ${\hat{y}}_{i}$ means the predicted CTR. $y_{i}\;$ indicates the true label.

### 3.2. Basic Model

**Figure 1:**
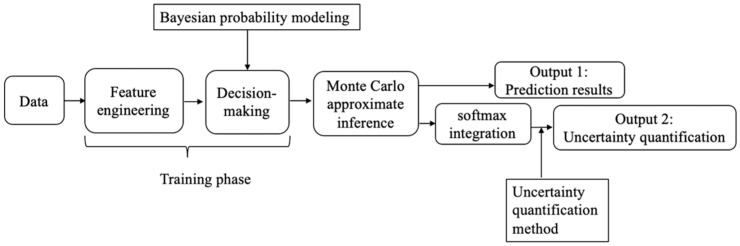
The construction idea of Bayesian deep learning.

The design idea of the Bayesian deep learning model is shown in Figure 1, which mainly includes the feature engineering phase, decision phase, and Monte Carlo reasoning phase, among which the feature engineering phase is completed by feature selection and the feature interaction module. Bayesian probability modeling is applied to fully connected neural networks. In this chapter, dropout regularization approximate Bayesian modeling is adopted.

**Figure 2:**
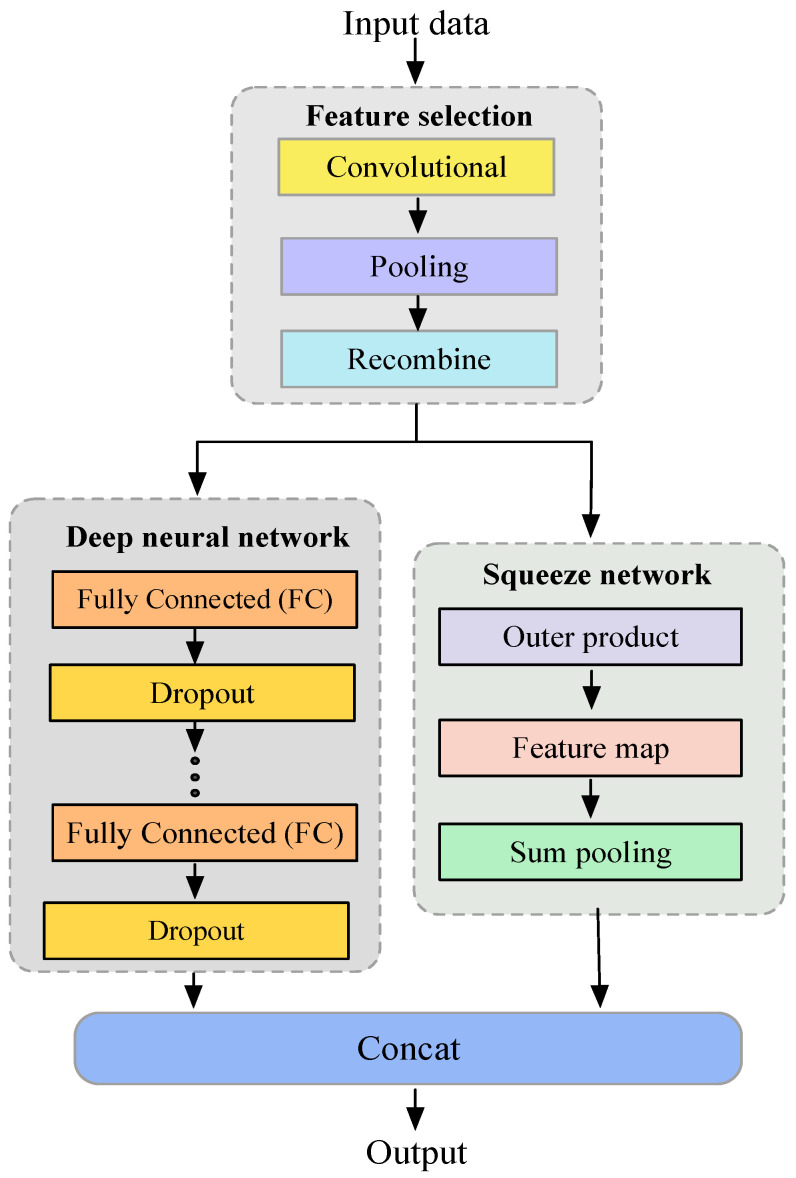
Diagram of the FiBDL model.

In this paper, we first exploit the strengths of the CNN to extract the neighbor feature interactions, while complementing it with a recombination layer to extract the global feature interactions. Then the squeezing network and DNN are applied to learn the high-order feature interactions in parallel. The diagram of the FiBDL is displayed in Figure 2.

(1) Feature selection. The feature selection module aims to identify useful local and global patterns to generate meaningful feature interactions. In the original feature space, meaningful feature interactions are always sparse. CNN has a parameter sharing and pooling mechanism, which can extremely decrease the number of parameters applied to discover important local patterns, which provides an idea for feature selection. Since it only produces neighbor feature interactions, many meaningful global feature interactions have been lost. So we use MLP to reorganize them to learn the global feature interaction.

Let $X=\left[x_{1,\ldots ,}x_{i}\right]$ represent the input. Let the output of the first convolutional layer be $C^{1}$. To obtain the neighbor feature interactions is to convolve the weight matrix ${\mathbb{C}}^{1}$ of the output feature map of the first convolution layer with non-linear activation functions. The convolutional layer is written as:

(2) $$C_{p,q,i}^{1}=\tan h\left(\overset{h^{1}}{\underset{j=1}{\sum }}x_{p+j-1,q,1}^{1}{\mathbb{C}}_{j,1,1,i}^{1}\right)$$

where $\;C_{:,:,i}^{1}$ is the i-th feature map of the first layer of the convolution, $h^{1}$ indicates the height of the convolution kernel, p, q denote the row and column indices of the feature map.

A max-pooling layer is applied after the convolutional layer to obtain the most important feature interactions. The output can be denoted as:

(3) $$S_{p,q,i}^{1}=\max\left(C_{p\cdot h_{p},q,i}^{1},\ldots ,C_{p\cdot h_{p}+h_{p}-1,q,i}^{1}\right)$$

where $h_{p}$ is the height of the pooling layers. It is worth noting that the input of the (i+1)-th convolutional layer is the pooling result of the i-th pooling layer: $S^{i}=x^{i+1}$.

$S^{1}$ includes neighbor feature interactions after going through the first convolutional layer and pooling layer. To obtain the global non-neighbor feature interactions, important new features are produced by recombining local neighbor feature interactions by the full connection layer.

(4) $$R^{1}=\tan h\left(W\cdot S^{1}+B\right)$$

where B denotes the bias and W means the weight matrix.

Performing the above process multiple times can generate new features.

(5) $$X=\left(R^{1},R^{2},\ldots ,R^{n}\right)$$

**Figure 3:**
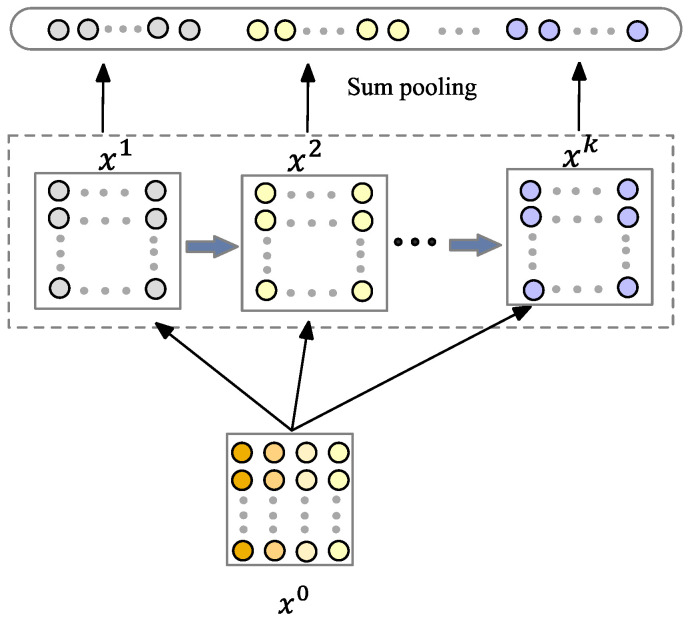
The structure of the squeeze network.

(2) Squeeze network. We have designed a squeeze network that is inspired by the CIN module in the xDeepFM model. The structure is shown in Figure 3. Its core is the summation and pooling of single hidden layer feature vectors, and the complexity of the model will not increase exponentially with the increase in the degree of interaction.

The features of the input and the hidden layer in the squeeze network are, respectively, organized into a matrix, denoted as $x^{0}$ and $x^{k+1}$. The neurons in each layer of the squeeze network are calculated based on the original input feature vector and the previous layer. The formula is:

(6) $$x_{h}^{k}=\overset{k-1}{\underset{i=1}{\sum }}\overset{m}{\underset{j=1}{\sum }}w_{i,j}^{k,h}\left(x_{i}^{0}\times x_{j}^{k-1}\right)$$

where $x^{k-1}$ represents the state of the previous layer, m is the number of feature fields.

**Figure 4:**
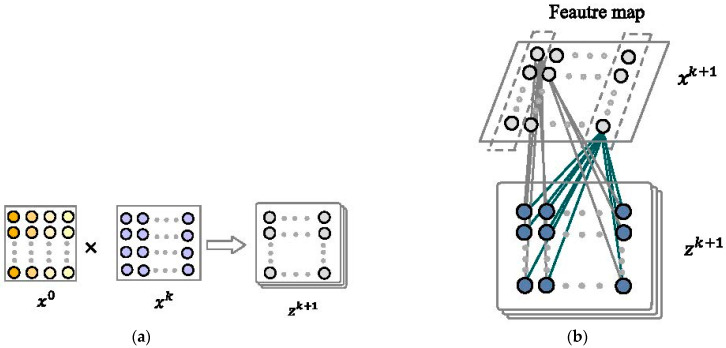
(**a**) Calculate the intermediate results. (**b**) Calculate the next hidden layer state.

The calculation of the hidden layer can divide it into two steps:

(1) The specific approach is the outer product of the vectors of each row of $x^{0}$ and $x^{k}$, resulting in a new vector, which is taken as the intermediate result $z^{k+1}$. The calculation process is shown in Figure 4a.

(2) The second step is to perform a convolution calculation on the result of the first step to obtain a new feature map result. Each convolution calculation will perform a convolution calculation on the entire feature map, as shown in Figure 4b.

The characteristics of the squeeze network are that the order of the feature interaction that is finally learned is decided by the number of layers of the network, and each hidden layer is connected to the output through a pooling operation, thus ensuring that the output unit can see feature interaction modes of a different order.

(3) Deep neural network. This module is composed of fully connected layers, which is a network with a multi-layer perceptron. There is a non-linear activation function between layers, a layer that is hidden from both input and output, and also needs to maintain a high degree of connectivity, which is determined by the weight of the network. The formula is as follows:

(7) $$y_{dnn}=\sigma \left(w_{z}\beta_{z}+b_{z}\right)$$

where $w_{z},b_{z}$ are the weight and bias. $\beta_{i}$ denotes the i-th hidden layer, σ is the activation function.

### 3.3. Uncertainty Quantification

The difference from the traditional Bayesian theory is that the Bayesian neural network introduces parameters into the prior distribution. To obtain the uncertainty estimation of the FiBDL model, the prior distribution is placed on the parameters of the deep neural network to determine the posterior distribution of parameters to achieve the best prediction.

The epistemic uncertainty is a measure of the uncertainty of model parameters. It derives from the limited representation ability of the observed value, which denotes the difference between the predicted value $\hat{y}$ and the true value y. The uncertainty of the model is quantified by placing a prior distribution over the model’s parameters ω and approximating the posterior by inference algorithm.

Given BDL with L layer has parameter $\omega ={\left\{w_{l},b_{l}\right\}}_{l=1}^{L}$. The prior distribution is the probability distribution of the model parameters independently from any observation. In Bayesian neural networks, it is usually assumed that the prior distribution p(ω) of parameter ω follows the standard Gaussian prior distribution: ω~N(0,1).

Upon determining an appropriate prior distribution based on prior knowledge, the model likelihood is p(Y|X,ω). According to Bayesian inference, the posterior of parameter ω can be calculated:

(8) $$p\left(\omega |D\right)=\frac{p\left(\omega \right)\cdot p(Y|X,\omega )}{p(Y|X)}$$

Under this posterior, if we have a new input instance $x^{*}$, the predicted output $y^{*}$ is given by

(9) $$p\left(y^{*}|x^{*},D\right)=\int^{\;}p\left(y^{*}|x^{*},\omega \right)\cdot p(\omega |D)d\omega$$

The target of Bayesian inference is to obtain the posterior p(ω|X,Y) of the model parameters for the given dataset. However, because of the complex non-conjugacy and non-linearity in the deep models, the posterior distribution is intractable. For this reason, different inference methods, such as the Markov chain Monte Carlo (MCMC) [39] and variational inference are proposed to approximate it. Gal et al. proposed approximate inference without changing the existing model architecture and proved that the posterior distribution can be approximated by the Monte Carlo dropout.

Specifically, this method defined an easy-to-evaluate distribution q(ω) to approximate the true posterior  p(ω|D). Then, it optimized the parameters of the defined distribution q(ω) to make it as close as possible to  p(ω|D). It is achieved by minimizing the KL divergence between the distributions p(ω|D) and  q(ω), which is denoted as:

(10) $$\begin{array}{ll} KL(q\left(\omega \right)||p(\omega |D))= & \int^{\;}q\left(\omega \right)\cdot log\frac{q\left(\omega \right)}{p(\omega |D)}d\omega \\ & =-\int^{\;}q\left(\omega \right)\cdot logp\left(Y|X,\omega \right)d\omega +KL(q\left(\omega \right)||p\left(\omega \right)) \end{array}$$

It has been proved in that the minimization of the KL divergence is equivalent to the maximization of the evidence lower bound. Maximizing it will result in the variational distribution q(ω)  being closer to the prior distribution and interpreting the data well. This approach has the advantage of replacing the integration problem with an optimization problem that maximizes the parameterized function. The evidence lower bound is equal to

(11) $${ℒ}_{VI}:=\int^{\;}q\left(\omega \right)\cdot logp\left(Y|X,\omega \right)d\omega -KL(q\left(\omega \right)||p\left(\omega \right))$$

the first term represents the *log*-likelihood expectation. It can be approximated by MC integration with a single sample ${\hat{\omega }}_{n}\sim q\left(\omega \right)$ to obtain an unbiased estimate $logp(y_{n}|x_{n},{\hat{\omega }}_{n})$. The basic concept of MC integration is to replace the integral with summation. The second term can be approximated by regularization, and its role is to avoid the model of over-fitting. Then, Equation (11) can be rewritten as:

(12) $${ℒ}_{VI}:=\overset{N}{\underset{n=1}{\sum }}\int^{\;}q\left(\omega \right)\cdot logp\left(y_{n}|x_{n},\omega \right)d\omega -KL(q\left(\omega \right)||p\left(\omega \right))$$

According to the approximate posterior  q(ω), the approximate predicted probability distribution of sample $x^{*}$ is:

(13) $$q(y^{*}|x^{*})\approx \int^{\;}p(y^{*}|x^{*},\omega )\cdot q\left(\omega \right)d\omega$$

Next, our predictive *log*-likelihood is approximated by MC integration of Equation (13). It is equivalent to performing T stochastic forward pass through the neural network and then averaging the results.

For CTR prediction, the general practice is to compress the model output by the Softmax function. The prediction can be carried out by approximating the predicted average in the following ways:

(14) $$E_{q\left(y^{*}|x^{*}\right)}\left(y^{*}\right)=p\left(y^{*}=c|x^{*},D\right)=\frac{1}{T}\overset{T}{\underset{t=1}{\sum }}p\left(y^{*}=c|x^{*},{\hat{\omega }}_{t}\right)\;\;\;\;\;\;\;\;\;\;\;\;\;\;\;\;\approx \frac{1}{T}\overset{T}{\underset{t=1}{\sum }}Softmax(f^{{\hat{\omega }}_{t}}\left(x^{*}\right))$$

where  c∈{0,1}, ${\hat{\omega }}_{t}\sim q_{\theta }\left(\omega \right)$,$\;q_{\theta }\left(\omega \right)$ represents the dropout distribution, $f^{\omega }\left(\cdot \right)$ denotes the prediction of the model.

In classification settings, uncertainty can be measured by predictive entropy $H\left(y^{*}|x^{*},D\right)$, which combines epistemic uncertainty and aleatoric uncertainty:

(15) $$H\left(y^{*}|x^{*},D\right)=MI\left(y^{*};\omega |x^{*},D\right)+E\left(H\left(y^{*}|x^{*},\omega \right)\right)$$

(16) $$H\left(y^{*}|x^{*},D\right)=-\underset{c}{\sum }p(y=c|x^{*},D)logp(y=c|x^{*},D)$$

For a new input $x^{*}$, The predictive entropy is approximate as:

(17) $$\hat{H}\left(y^{*}|x^{*},D\right)=-\underset{c}{\sum }\left(\frac{1}{T}\underset{t}{\sum }p(y=c|x^{*},{\hat{\omega }}_{t})log(\frac{1}{T}\underset{t}{\sum }p(y=c|x^{*},{\hat{\omega }}_{t})\right)$$

In a Bayesian neural network, T MC samples are used to take the average entropy of a single input to capture the aleatoric uncertainty related to the data. The mutual information (MI) between the parameter ω and the output $y^{*}$ is used to quantify the epistemic uncertainty

(18) $$E\left(\hat{H}\left(y^{*}|x^{*},\omega \right)\right)=-\frac{1}{T}\underset{t,c}{\sum }p\left(y=c|x^{*},{\hat{\omega }}_{t}\right)logp\left(y=c|x^{*},{\hat{\omega }}_{t}\right)$$

(19) $$\begin{array}{ll} \hat{MI}\left(y^{*};\omega |x^{*},D\right) & =\hat{H}\left(y^{*}|x^{*},D\right)-E\left(\hat{H}\left(y^{*}|x^{*},\omega \right)\right)\\ & =-\sum_{c}\left(\frac{1}{T}\sum_{t}p\left(y=c|x^{*},{\hat{\omega }}_{t}\right)log(\frac{1}{T}\sum_{t}p\left(y=c|x^{*},{\hat{\omega }}_{t}\right)\right)\\ & +\frac{1}{T}\sum_{t,c}p\left(y=c|x^{*},{\hat{\omega }}_{t}\right)logp\left(y=c|x^{*},{\hat{\omega }}_{t}\right) \end{array}$$

## 4. Experimental Results

Our main work is to construct a prediction model and measure the uncertainty. In this section, we validate the FiBDL model from two aspects. The first one is to illustrate the superiority of the model in CTR prediction by comparing it with several benchmark models. The second is to quantify the uncertainty of the proposed model in its predictions.

### 4.1. Experimental Settings

(1) Datasets

(1) Taobao dataset is a dataset of CTR prediction about display ads, which is displayed on the website of Taobao. The dataset consists of two parts: user_profile and ad_features.

(2) Avazu dataset is published in the CTR prediction contest on Kaggle in 2014. It contains four types of attributes: user, website, advertisement, and time. Each click sample has 24 data fields, including 22 category features.

(3) ICME dataset is utilized to predict whether users will finish watching and like videos. Each click sample has two dense features and nine sparse fields.

Following shuffling, all of the datasets are further randomly divided into train and test sets. We randomly placed 80% of the sample data in the training set and 20% in the test set.

(2) Evaluation metrics

In this paper, we use Logloss and RMSE, two metrics to measure the prediction performance of all models.

Logloss is a criterion widely applied for classification problems. It balances the difference between the predicted value and the true value. Its definition is as in Equation (1).

RMSE is a criterion of the deviation between the predicted value and the true value. As a metric for uncertainty in this article, the RMSE is denoted as:

(20) $$RMSE=\sqrt{\frac{1}{N}\overset{N}{\underset{i=1}{\sum }}{\left({\hat{y}}_{i}-y_{i}\right)}^{2}}$$

(3) Baselines

The FiBDL model is compared with the following models, each model is briefly described as follows:

- LR. The LR model can only learn linear combinations of features and lacks the ability to obtain feature interactions;
- FM. The FM model only can learn second-order feature interaction information but cannot model high-order feature interactions;
- AFM. The AFM added an attention mechanism to the FM model to obtain feature weights of effective features;
- FNN. The FNN model placed FM pre-trained low-dimensional features on DNN, enabling it to generate high-order features automatically;
- WDL. The WDL adopted a dual-path structure, which joins the generalization of the deep model with the memory of the wide linear model;
- xDeepFM. The xDeepFM proposed CIN for vector-wise feature interaction that can obtain explicit and implicit high-order feature interactions simultaneously;
- AutoInt. The AutoInt model employed a multi-head self-attention network to attain explicit higher-order feature interactions;
- FiBiNET. The FiBiNET adopted SENET to learn the importance of features, and learn feature interactions by a bilinear function;
- DIFM. The DIFM model utilized fully-connected layers and multi-head self-attention networks, respectively, to obtain feature interactions from bit-wise and vector-wise levels;
- DeepFEFM [40]. The DeepFEFM model employed a field-embedding factorization machine to learn a symmetric matrix embedding for each field pair, as well as a common single-vector embedding for each feature;
- DCN V2 [41]. The DCN V2 reduced the dimension of the parameter matrix by low-rank matrix decomposition and effectively learned feature interactions;
- MoFM. The MoFM model combined LR, FM, and self-attention networks to obtain both low- and high-order feature interactions;
- XCrossNet [42]. The extreme cross network (XCrossNet) model proposed a three-stage model, which is used to model the interaction of dense and sparse features, respectively, and interact with each other at the concatenation layer, and finally use MLP to capture the feature interaction.

### 4.2. Performance Comparison

Compare the prediction performance of the FiBDL model with the existing classical efficient model described in the previous section. The performance of all models is shown in Table 1, The observation results are obtained as follows.

The performance of LR is worse than other models, which illustrates the ability of the performance of both factorization models and deep neural network-based models. FM and AFM models can model second-order feature interactions to improve prediction accuracy. AFM significantly outperforms FM on all datasets, which is probably attributed to the performance of the attention mechanism in feature learning. Models, such as FNN, xDeepFM, etc., have achieved further performance improvements by combining FM and neural networks into a framework. The reason is that neural networks help FM-based models draw out helpful information for the prediction task. Furthermore, both FiBiNET and DIFM can measure feature importance in the feature learning stage. Models that combine core architectural components that learn explicit or implicit feature interactions with deep neural network components are beneficial for improving prediction performance.

Experimental results show that compared with the classical shallow prediction methods (LR, FM), the FiBDL has an advantageous position in prediction performance. The improvement stems from the fact that the FiBDL model learns high-order feature interaction information. The performance of the FiBDL model significantly outperforms the best and most advanced models. The performance metric Logloss is decreased by about 3.25%, 11.99%, and 1.81% compared to the best baseline method on all datasets, respectively. The reason may be that we completed the feature selection in the initial stage, which pruned redundant feature information. This is useful and positive for feature learning. Learning both implicit and explicit higher-order feature interactions simultaneously plays an important role in prediction tasks and is useful for improving performance.

Of the three datasets, FiBDL is about 1.95%, 9.77%, and 0.55% lower than the best performing model on the RMSE criterion, respectively. According to the results of Logloss and RMSE on the three datasets, the superiority of the proposed FiBDL model in the CTR prediction task is verified.

### 4.3. Feasibility of Using MC Dropout

To verify the effectiveness of using Monte Carlo with dropout to model uncertainty, we compare the FiBDL model with the basic model without dropout. According to Table 2, models using the MC dropout perform well on three datasets in terms of prediction performance. From the Logloss aspect, the model using the MC dropout model achieves a performance improvement of 0.42%, 0.22%, and 0.19% on these three datasets of the basic model, respectively. The Logloss criterion shows the effect of the model prediction. For the RMSE metric, the model FiBDL with the MC dropout achieves performance improvements of 0.26%, 0.1%, and 0.07%, respectively. Among them, there is a more minor improvement in the ICME dataset. This set of experimental results proves that the use of the Monte Carlo with dropout can improve the prediction accuracy. At the same time, it can reduce network overfitting, and achieve the effectiveness of the prediction.

### 4.4. Ablation Study

To verify the validity of each part in the FiBDL model and to better understand their relative importance, in this set of experiments, one part is removed and the rest are kept constant.

- No feature selection (FS): removing the feature selection module means that meaningful features cannot be selected for feature interaction;
- No squeeze network (SN): removing the squeeze network indicates that the model cannot learn higher-order feature interactions explicitly;
- No DNN: removing the deep neural network, then the model cannot model higher-order feature interactions implicitly.

(1) Prediction performance

**Figure 5:**
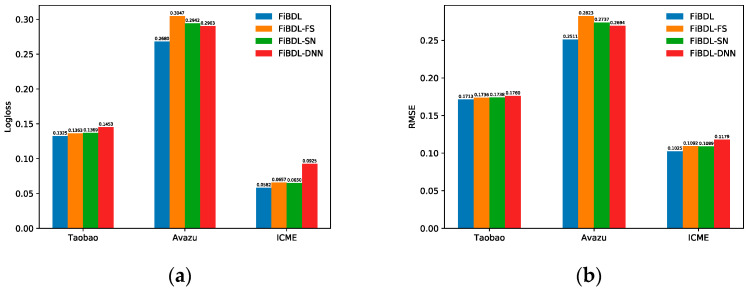
Performance of the different components of the FiBDL model. (**a**) The Logloss of different variants, (**b**) The RMSE of different variants.

Observe the two metrics that measure the CTR prediction performance, as can be seen in Figure 5:

Removing the feature selection module, the performance of the model on the Avazu dataset degrades significantly. It increased by about 3.67% and 3.12% in Logloss and RMSE, respectively. For the other two datasets, there is 0.38% and 0.75% heightened in Logloss (0.23% and 0.67% by RMSE). It demonstrates that the selection of the input data before feature interaction can improve the prediction performance.

Once DNN was deleted, the Logloss of the model on all datasets was significantly reduced by about 1.28%, 2.23%, and 3.43%, respectively. The performance degradation was significant on three datasets. It proved that the modeling of high-order feature interaction implicitly by DNN has a considerable influence on the model.

Following the removal of the squeeze network, the performance of the model on the Avazu dataset has remarkably fallen. The Logloss on the remaining two datasets is not significant. The RMSE on the three datasets has increased approximately by 0.25%, 2.26%, and 0.64%, respectively. It demonstrates the effectiveness of squeeze networks in learning explicit higher-order feature interactions. It can be seen that FiBDL outperforms all ablation methods. This validates that any component of the FiBDL model proposed is critical for prediction performance.

(2) Uncertainty Quantification

**Figure 6:**
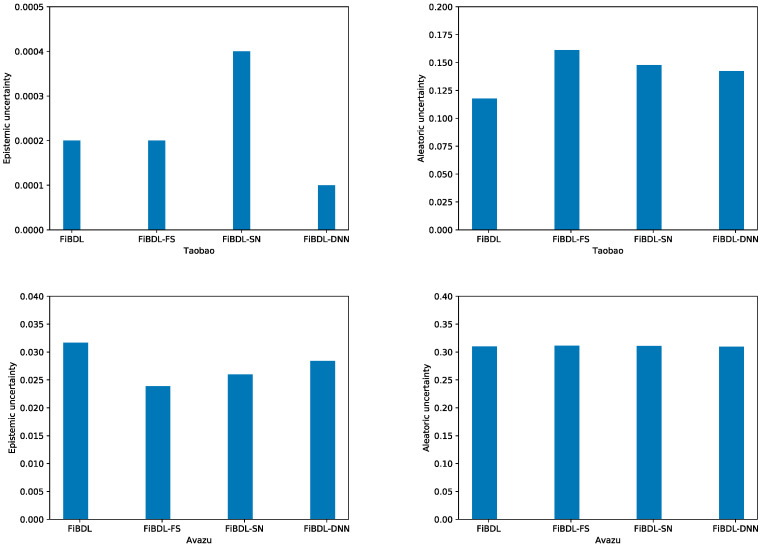

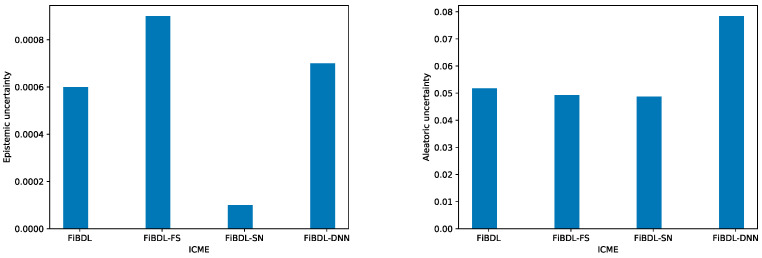
Uncertainty of the different variants of the FiBDL model.

As Figure 6 shows, the ability of the model to quantify uncertainty fluctuates when we remove any components from the FiBDL model. On the three datasets, the quantified aleatoric uncertainty shows a small bias, which stems from the constant size of the experimental dataset. The aleatoric uncertainty is data-dependent. Epistemic uncertainty is tightly correlated with model parameters, and the ability to quantify this part will be affected if any component of the model is deleted.

### 4.5. Influence of the Training Data Size

To examine how the predictive performance and two types of the uncertainty of the FiBDL model vary as the training data increases, we use 20%, 40%, 60%, 80%, and 100% of the training data, respectively, to train the FiBDL model.

**Figure 7:**
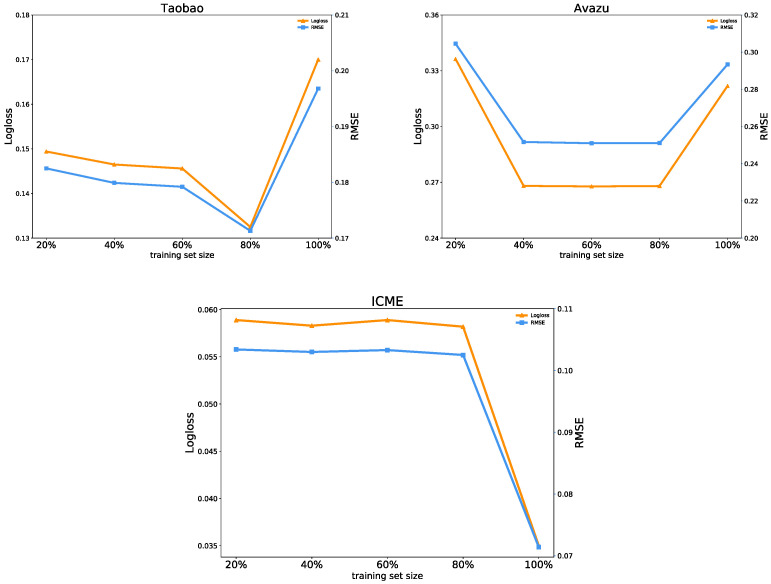
Prediction performance with the training data size.

As shown in Figure 7, as the training data increase, the prediction performance of the FiBDL model on the Taobao and Avazu datasets first increases and then decreases. When the training set accounts for 80% of the dataset, the prediction performance on the Taobao dataset achieves the best. On the Avazu dataset, the prediction performance is optimal when the training set accounts for 60%. The performance of the FiBDL model on the ICME dataset initially fluctuates less, and when the training data reach 100%, the model has enough training, and the prediction performance outperforms well.

**Figure 8:**
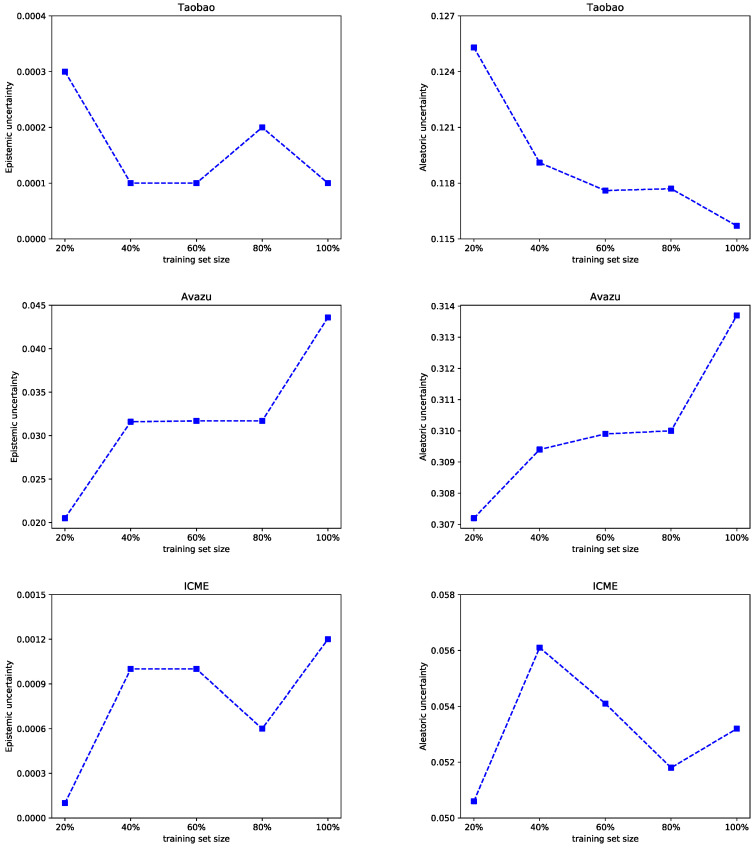
Uncertainty with the training data size.

Figure 8 shows the relationship between the two classes of uncertainty and the percentage of the training data applied during training. We already know that epistemic uncertainty is caused by the parameters of the model and is caused by incomplete training. Aleatoric uncertainty originated from the data noise, and such uncertainty cannot be eliminated. With the training data increasing, the aleatoric uncertainty of the FiBDL model on the Taobao dataset decreased. Epistemic uncertainty showed less fluctuation. On the Avazu dataset, both uncertainties declined. When the proportion of the training dataset increased from 80% to 100%, the increase rate was much faster, which may be on account of the homogeneous nature of the Avazu dataset itself.

### 4.6. Hyper-Parameter Study

(1) Effect of the Dropout Ratio

Dropout is a regularization technique that prevents overfitting. It refers to temporarily dropping neurons from a deep learning network at a given probability during training. In this paper, we conduct experiments with a dropout ratio ranging from {0.05, 0.1, 0.15, 0.2, 0.25, 0.3}.

**Figure 9:**
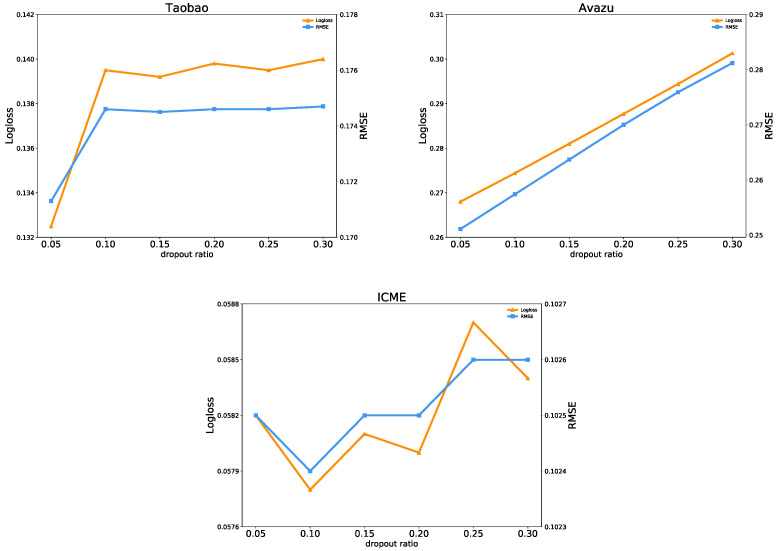
Prediction performance with the dropout ratio.

Figure 9 displays the influence of the dropout ratio on the prediction performance of FiBDL. As the dropout ratio grows, the performance of FiBDL on the three datasets decreases to a large extent. On the Avazu dataset, the values of Logloss and RMSE have been increasing. This means that with the dropout ratio increasing, the prediction performance has been decreasing by 3.33% and 3.01%, respectively. On the other two datasets, the values of Logloss and RMSE fluctuate. On the Taobao dataset, the performance in terms of Logloss and RMSE declines sharply with the dropout ratio increasing and then fluctuates slightly. On the ICME dataset, when the dropout ratio is 0.1, the optimal performance is achieved on both metrics. In this experiment, the dropout ratio is fixed at 0.05.

**Figure 10:**
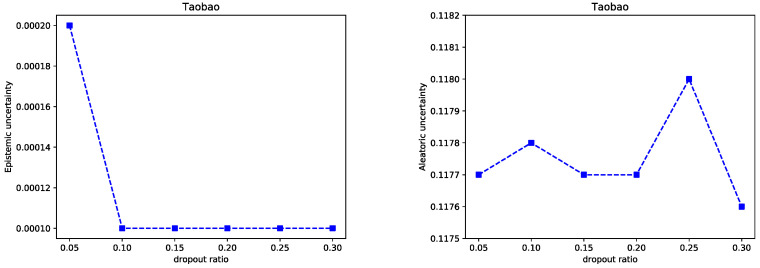

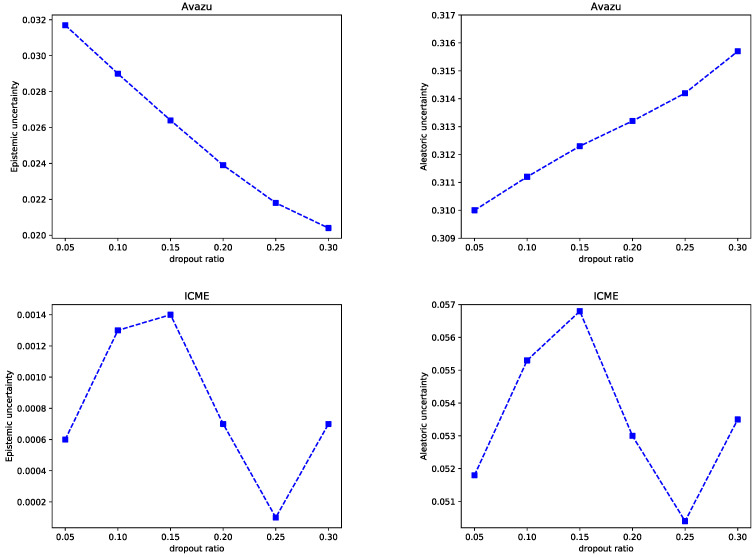
Uncertainty with different dropout ratios.

Figure 10 shows the relationship between the uncertainty of the prediction model and the dropout ratio. It can be found that, on the Avazu dataset, the quantified epistemic uncertainty decreases steadily as the dropout rate increases, while the aleatoric uncertainty increases steadily. The reason is that as the dropout value increases, the model will be more robust, equivalent to dropping some features. Epistemic uncertainty is related to model parameters, and aleatoric uncertainty is data-based. On the Taobao dataset, the quantification of aleatoric uncertainty is sensitive to the dropout ratio. It may be that when training the network, dropout sampling is performed so that the model does not rely too much on certain features, even if they are real. On the ICME dataset, both uncertainties have an upward trend and then drop sharply with the increasing dropout ratio, reaching the optimal state when the dropout value is 0.25.

(2) Effect of the Network Depth

In the deep part, adding hidden layers can better separate the features of the data. The number of network layers is changed, and we observe how the model performance and uncertainties change with the increase of the network layers.

**Figure 11:**
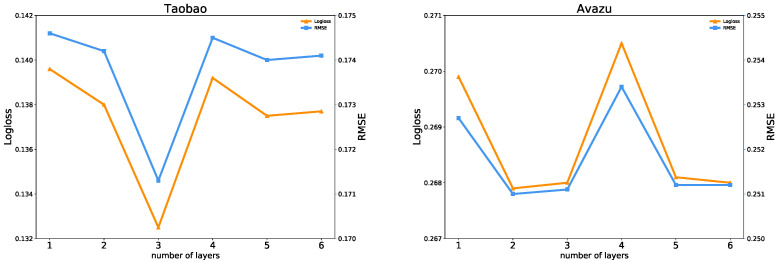

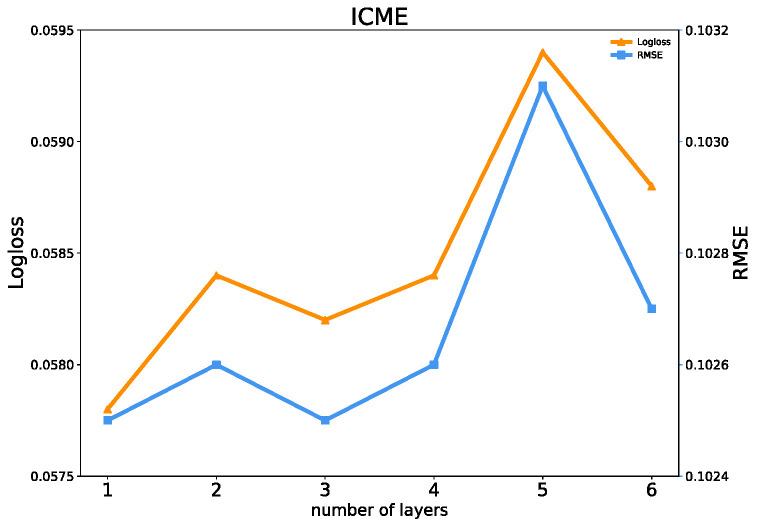
Prediction performance w.r.t. different depths in the DNN.

Figure 11 shows the influence of the network depth in the FiBDL model. In particular, we study the network depth in the range {1, 2, 3, 4, 5, 6}. We observed that the FiBDL model on the Taobao dataset has the best prediction performance when the network depth is 3. When the number of layers is less than 3, the model performance is positively correlated with the network depth, and the predictive performance decreases as the network depth increases. When the number of layers is 1 or 2, the FiBDL model has the best prediction performance on the ICME and Avazu datasets. The results illustrate that excessive hidden layers cannot supply great improvement for the FiBDL model, so it is essential to select reasonable network depths.

**Figure 12:**
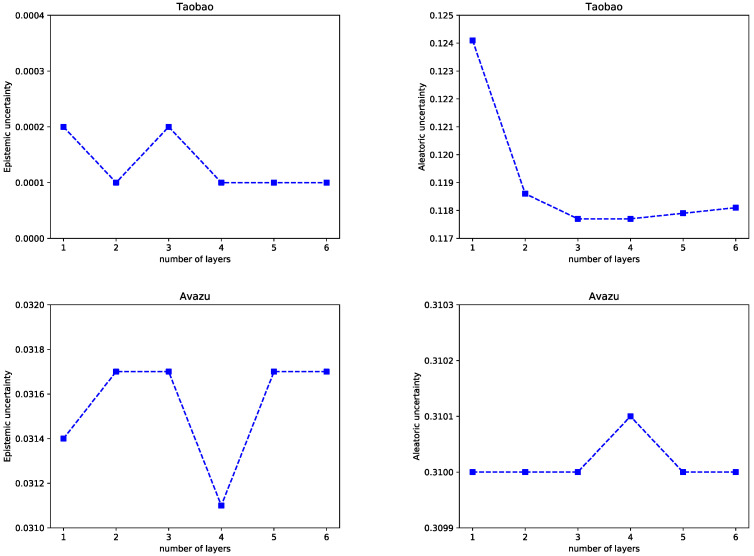

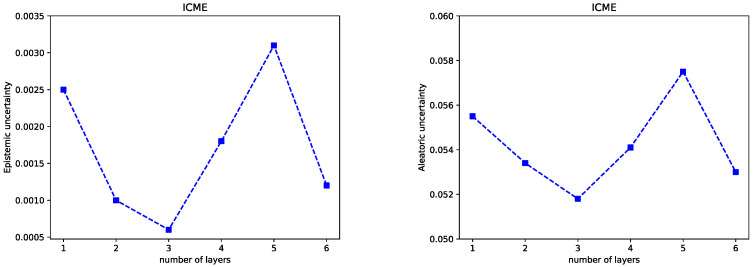
Uncertainty w.r.t. different depths in the DNN.

By increasing the depth of the neural network, the number of MC dropout layers of the FiBDL model will also increase. The results are shown in Figure 12. In the ICME dataset, both uncertainties of the model decrease when the network depth is less than 3. When the network depth is 3, the uncertainty reaches the minimum. On the Taobao dataset, the fluctuation of epistemic uncertainty is small. The aleatoric uncertainty achieves the minimum value when the number of layers is 3. The deviation of the aleatoric uncertainty of the FiBDL model on the Avazu dataset is very small. Based on comprehensive considerations, a 3-layer network is appropriate.

(3) Effect of the Training Epoch

The experiments on the training epoch of the model are tested in this section. Experiments were performed on the same randomly generated train-test splits of the data. Experiments are on the same training-test dataset.

**Figure 13:**
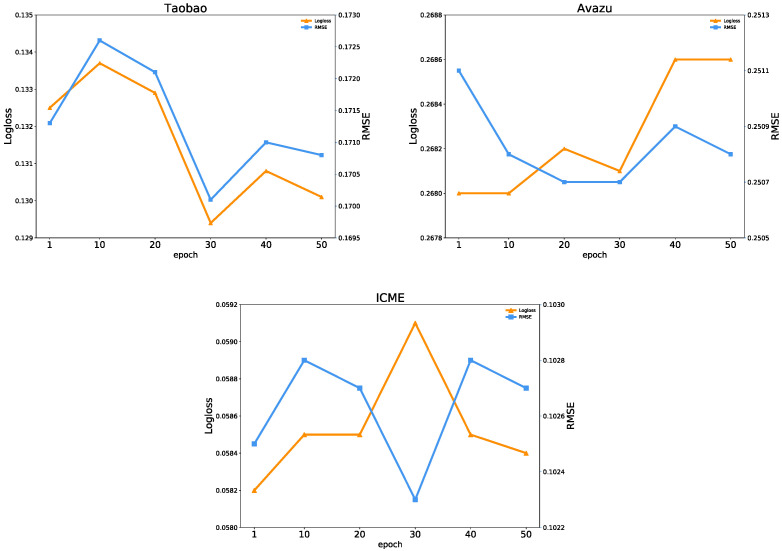
Prediction performance with different epochs.

Figure 13 shows that the prediction performance and model uncertainty of the FiBDL model on the three datasets change significantly with the increase of the training epoch. On the Taobao dataset, the best prediction performance is achieved when the training epoch is 30. On the Avazu dataset, as the training epoch increases, the two prediction performance metrics of the model do not reach the optimum simultaneously. On the ICME dataset, the training epochs of the original experiment are the settings when the performance is optimal.

**Figure 14:**
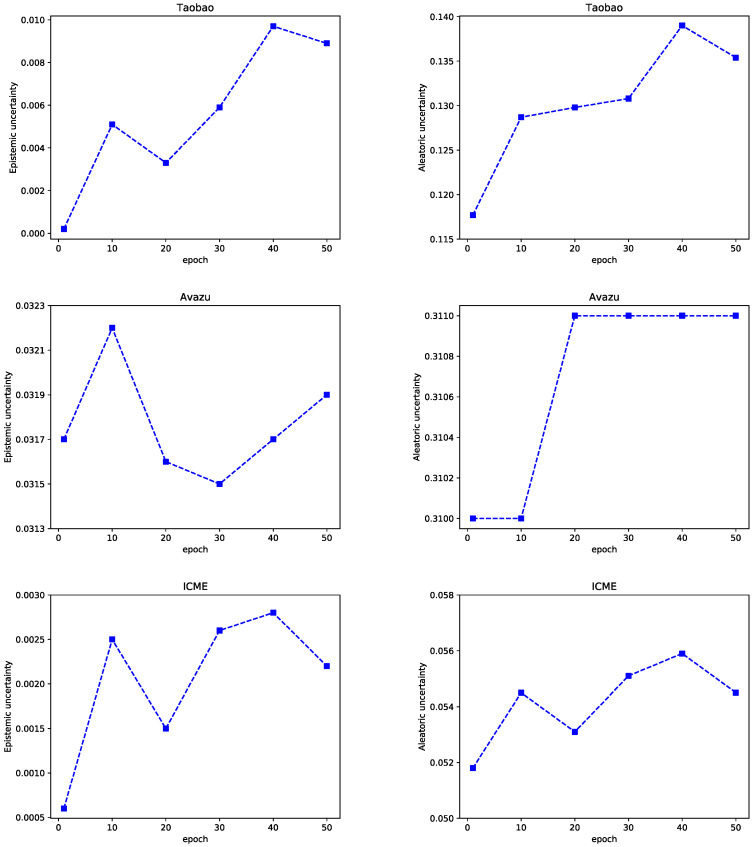
Uncertainty with different epochs.

The uncertainty results are shown in Figure 14. For the Taobao dataset, with the increase of epoch, the two uncertainties show an overall trend of increasing. When the training epoch is 40, the two uncertainties are the largest. Among them, the aleatoric uncertainty increases greatly. For the Avazu dataset, when the training epoch increases from 10 to 20, the epistemic uncertainty decreases rapidly. Aleatoric uncertainty increases and then remains unchanged. For the Taobao and ICME datasets, the uncertainty is minimal when the training epoch is set as 1, the best model for each type of uncertainty is obtained for both datasets.

## 5. Conclusions

Aiming at the uncertainty problem in the CTR prediction model, we propose a CTR prediction model based on Bayesian deep learning. To improve the representation of the features used in the model, we build a prediction structure that combines feature selection and feature interaction. Feature selection module is used to select meaningful global feature interactions, squeeze network, and DNN in parallel and is used to model higher-order feature interactions. we train a dropout network and use dropout to obtain prediction Monte Carlo samples at test time, and then use information entropy to quantify the uncertainty. Bayesian deep learning architecture can reliably estimate the uncertainty and improve the robustness of the model by introducing uncertainty into the parameters of the neural network. Experimental results show that this model can provide reliable prediction and effective uncertainty estimation.

## Figures and Tables

**Table 1:** Performance for the different models.

Model	Taobao		Avazu		ICME	
	Logloss	RMSE	Logloss	RMSE	Logloss	RMSE
LR FM AFM	0.3419 0.3387 0.3099	0.2938 0.2912 0.2744	0.4263 0.4241 0.421	0.3646 0.3633 0.3614	0.1646 0.1316 0.1246	0.1618 0.14 0.134
FNN	0.2603	0.2398	0.4007	0.3559	0.089	0.1249
WDL	0.2215	0.2095	0.3948	0.352	0.0865	0.1204
xDeepFM	0.165	0.1961	0.39	0.35	0.0812	0.108
AutoInt	0.2162	0.214	0.3933	0.3511	0.0834	0.1134
FiBiNET	0.1761	0.1938	0.3936	0.3514	0.079	0.1115
DIFM	0.1792	0.2083	0.4175	0.3596	0.0916	0.115
DCN V2 MoFM DeepFEFM XCrossNet	0.1661 0.1979 0.2254 0.1876	0.1908 0.2003 0.2164 0.2	0.3879 0.3929 0.3993 0.3938	0.3488 0.3512 0.3547 0.3511	0.094 0.078 0.0802 0.0763	0.113 0.109 0.1103 0.1149
FiBDL	0.1325	0.1713	0.268	0.2511	0.0582	0.1025

**Table 2:** Performance with and without dropout.

Method	Taobao		Avazu		ICME	
	Logloss	RMSE	Logloss	RMSE	Logloss	RMSE
With MC dropout Without dropout	0.1325 0.1367	0.1713 0.1739	0.268 0.2702	0.2511 0.2521	0.0582 0.0601	0.1025 0.1032

## Data Availability

The datasets used and analyzed during the current study are available from the corresponding author upon reasonable request.

## References

[1] McMahan H.B., Holt G., Sculley D., Young M., Ebner D., Grady J., Nie L., Phillips T., Davydov E., Golovin D. Ad click prediction: A view from the trenches. Proceedings of the 19th ACM SIGKDD International Conference on Knowledge Discovery and Data Mining (ACM).

[2] Rendle S. Factorization machines. Proceedings of the 2010 IEEE International Conference on Data Mining ICDM.

[3] Xiao J., Ye H., He X., Zhang H., Wu F., Chua T.S. Attentional factorization machines: Learning the weight of feature interactions via attention networks. Proceedings of the Twenty-Sixth International Joint Conference on Artificial Intelligence.

[4] Qu Y., Cai H., Ren K., Zhang W., Yu Y., Wen Y., Wang J. Product-based neural networks for user response prediction. Proceedings of the 2016 IEEE 16th International Conference on Data Mining (ICDM).

[5] Wang R., Fu G., Fu B., Wang M. Deep & cross network for ad click predictions. Proceedings of the 2017 AdKDD TargetAd—Conjunction with 23rd ACM SIGKDD International Conference on Knowledge Discovery and Data Mining, KDD.

[6] Guo H., Tang R., Ye Y., Li Z., He X. DeepFM: A factorization-machine based neural network for CTR prediction. Proceedings of the IJCAI International Joint Conferences on Artificial Intelligence.

[7] Zhou G., Song C., Zhu X., Fan Y., Zhu H., Ma X., Yan Y., Jin J., Li H., Gai K. Deep Interest Network for Click-Through Rate Prediction. Proceedings of the 24th ACM SIGKDD International Conference on Knowledge Discovery & Data Mining.

[8] Zhou G., Mou N., Fan Y., Pi Q., Bian W., Zhou C., Zhu X., Gai K. Deep interest evolution network for click-through rate prediction. Proceedings of the 33rd AAAI Conference on Artificial Intelligence, AAAI 2019.

[9] Yan C., Li X., Chen Y., Zhang Y. (2021). JointCTR: A joint CTR prediction framework combining feature interaction and sequential behavior learning. Appl. Intell..

[10] Maher M., Ngoy P.M., Rebriks A., Ozcinar C., Cuevas J., Sanagavarapu R., Anbarjafari G. (2022). Comprehensive Empirical Evaluation of Deep Learning Approaches for Session-Based Recommendation in E-Commerce. Entropy.

[11] Wang X., Dong H., Han S. (2020). Click-Through Rate Prediction Combining Mutual Information Feature Weighting and Feature Interaction. IEEE Access.

[12] Huang T., Zhang Z., Zhang J. Fibinet: Combining feature importance and bilinear feature interaction for click-through rate prediction. Proceedings of the RecSys 2019—13th CM Conference On Recommender Systems.

[13] Lu W., Yu Y., Chang Y., Wang Z., Li C., Yuan B. A dual input-aware factorization machine for CTR prediction. Proceedings of the IJCAI International Joint Conference on Artificial Intelligence.

[14] Gal Y., Ghahramani Z. Dropout as a Bayesian approximation: Representing model uncertainty in deep learning. Proceedings of the 33rd International Conference on Machine Learning ICML.

[15] Gai K., Zhu X., Li H., Liu K., Wang Z. (2017). Learning Piece-wise Linear Models from Large Scale Data for Ad Click Prediction. arXiv.

[16] He X., Pan J., Jin O., Xu T., Liu B., Xu T., Shi Y., Atallah A., Herbrich R., Bowers S. Practical lessons from predicting clicks on ads at Facebook. Proceedings of the 20th ACM SIGKDD International Conference on Knowledge Discovery and Data Mining.

[17] Juan Y., Zhuang Y., Chin W.S., Lin C.J. Field-aware factorization machines for CTR prediction. Proceedings of the RecSys 2016—ACM Conference on Recommender Systems.

[18] Blondel M., Fujino A., Ueda N., Ishihata M. (2016). Higher-order factorization machines. Advances in Neural Information Processing Systems.

[19] Pan J., Xu J., Ruiz A.L., Zhao W., Pan S., Sun Y., Lu Q. FWFM:Field-weighted factorization machines for click-through rate prediction in display advertising. Proceedings of the the 2018 World Wide Web Conference.

[20] Yan C., Chen Y., Wan Y., Wang P. (2021). Modeling low- and high-order feature interactions with FM and self-attention network. Appl. Intell..

[21] Zhang W., Du T., Wang J. (2016). Deep learning over Multi-Field categorical Data—A case study on user response prediction. Advances in Information Retrieval.

[22] Karatzoglou A., Hidasi B. Wide & Deep Learning for Recommender Systems. Proceedings of the the Eleventh ACM Conference on Recommender Systems.

[23] Lian J., Chen Z., Zhou X., Xie X., Zhang F., Sun G. xDeepFM: Combining explicit and implicit feature interactions for recommender systems. Proceedings of the 24th ACM SIGKDD International Conference on Knowledge Discovery & Data Mining.

[24] Liu M., Cai S., Lai Z., Qiu L., Hu Z., Ding Y. (2021). A joint learning model for click-through prediction in display advertising. Neurocomputing.

[25] Feng Y., Lv F., Shen W., Wang M., Sun F., Zhu Y., Yang K. Deep session interest network for click-through rate prediction. Proceedings of the IJCAI International Joint Conference on Artificial Intelligence.

[26] Song W., Shi C., Xiao Z., Duan Z., Xu Y., Zhang M., Tang J. Autoint: Automatic feature interaction learning via self-attentive neural networks. Proceedings of the 28th ACM International Conference on Information and Knowledge Management.

[27] Song K., Huang Q., Zhang F., Lu J. (2021). Coarse-to-fine: A dual-view attention network for click-through rate prediction. Knowl.-Based Syst..

[28] Li D., Hu B., Chen Q., Wang X., Qi Q., Wang L., Liu H. (2021). Attentive capsule network for click-through rate and conversion rate prediction in online advertising. Knowl.-Based Syst..

[29] Wang J., Niu T., Du P., Yang W. (2020). Ensemble probabilistic prediction approach for modeling uncertainty in crude oil price. Appl. Soft Comput..

[30] Liu Y., Qin H., Zhang Z., Pei S., Jiang Z., Feng Z., Zhou J. (2020). Probabilistic spatiotemporal wind speed forecasting based on a variational Bayesian deep learning model. Appl. Energy.

[31] Sun M., Zhang T., Wang Y., Strbac G., Kang C. (2019). Using Bayesian Deep Learning to Capture Uncertainty for Residential Net Load Forecasting. IEEE Trans. Power Syst..

[32] Xiao Y., Wang W.Y. Quantifying uncertainties in natural language processing tasks. Proceedings of the 33rd AAAI Conference on Artificial Intelligence, AAAI 2019.

[33] Hernández S., López J.L. (2020). Uncertainty quantification for plant disease detection using Bayesian deep learning. Appl. Soft Comput. J..

[34] Ghoshal B., Tucker A. (2020). Estimating Uncertainty and Interpretability in Deep Learning for Coronavirus (COVID-19) Detection. arXiv.

[35] Abdar M., Samami M., Mahmoodabad S.D., Doan T., Mazoure B., Hashemifesharaki R., Liu L., Khosravi A., Acharya U.R., Makarenkov V. (2021). Uncertainty quantification in skin cancer classification using three-way decision-based Bayesian deep learning. Comput. Biol. Med..

[36] Wang X., Kadıo S. (2021). Modeling uncertainty to improve personalized recommendations via Bayesian deep learning. Int. J. Data Sci. Anal..

[37] Zheng R., Zhang S., Liu L., Luo Y., Sun M. (2020). Uncertainty in Bayesian deep label distribution learning. Appl. Soft Comput..

[38] Jin X.B., Gong W.T., Kong J.L., Bai Y.T., Su T.L. (2022). A Variational Bayesian Deep Network with Data Self-Screening Layer for Massive Time-Series Data Forecasting. Entropy.

[39] Hao W., Yeung D.Y. (2016). Towards Bayesian Deep Learning: A Framework and Some Existing Methods. IEEE Trans. Knowl. Data Eng..

[40] Pande H. (2020). Field-Embedded Factorization Machines for Click-Through Rate Prediction. arXiv.

[41] Wang R., Shivanna R., Cheng D., Jain S., Lin D., Hong L., Chi E. (2021). DCN V2: Improved Deep & Cross Network and Practical Lessons for Web-Scale Learning to Rank Systems.

[42] Yu R., Ye Y., Liu Q., Wang Z., Yang C. (2021). XCrossNet: Feature Structure-Oriented Learning for Click-Through Rate Prediction. Advances in Knowledge Discovery and Data Mining.

